# A case report of leptospirosis complicated by severe pulmonary hemorrhage treated with venovenous extracorporeal membrane oxygenation

**DOI:** 10.3389/fmed.2025.1696005

**Published:** 2025-11-12

**Authors:** Li Wen Yang, Xiao Gang Tang, Bin Xiong, Yunli Zhang

**Affiliations:** People's Hospital of Guangxi Zhuang Autonomous Region, Nanning, China

**Keywords:** leptospirosis, pulmonary hemorrhage, venovenous extracorporeal membrane oxygenation (VV-ECMO), acute respiratory distress syndrome (ARDS), lymphoplasmacyte exchange (LPE)

## Abstract

Leptospirosis is a zoonotic disease with diverse clinical manifestations, and its severe form can lead to life-threatening complications such as pulmonary hemorrhage. We present a novel case of a 57-year-old woman with leptospirosis who developed severe pulmonary hemorrhage and acute respiratory distress syndrome (ARDS) and was successfully managed with early venovenous extracorporeal membrane oxygenation (VV-ECMO), minimal anticoagulation, and lymphoplasmacyte exchange (LPE). This case highlights the importance of early ECMO initiation, individualized anticoagulation, and immunomodulatory therapy in improving outcomes for patients with leptospiral pulmonary hemorrhage syndrome (LPHS), a condition with mortality exceeding 50%. To our knowledge, this is the first reported case combining VV-ECMO with LPE in LPHS, offering a new therapeutic paradigm for critically ill patients at the intersection of infection and autoimmunity.

## Introduction

Leptospirosis is a globally prevalent zoonosis caused by pathogenic Leptospira species. It is primarily transmitted to humans through contact with water or soil contaminated by the urine of infected animals, including rodents and livestock (1, 2). The clinical spectrum of leptospirosis ranges from a mild, flu-like illness to severe forms involving multiple organ systems, such as the lungs, liver, kidneys, and central nervous system (3). Pulmonary involvement in leptospirosis can present as mild pneumonia or, in severe cases, as massive pulmonary hemorrhage, which is associated with a high mortality rate (4, 5). While ECMO has been used in severe leptospirosis, the combination with immunomodulatory techniques such as LPE remains unexplored.

## Case presentation

### Initial presentation

**Figure 1:**
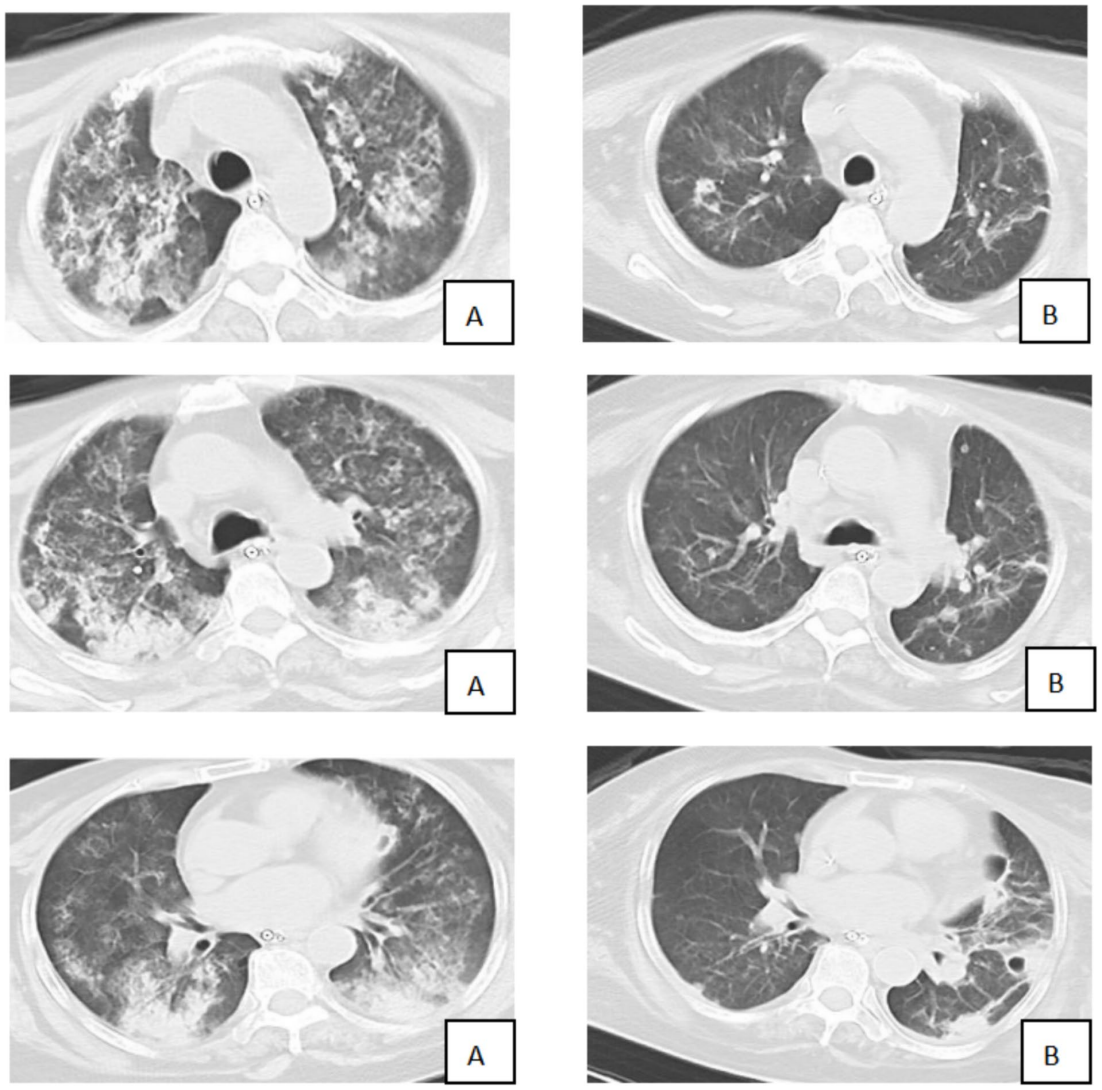
Comparison of chest CT before and after treatment (**A**: 1st August; **B**: 8th August).

A 57-year-old female farmer was admitted to our hospital with a 3-day history of fever, fatigue, myalgia, and headache. The patient denied recent travel outside the local area but reported frequent work in rice paddy fields. On admission, the patient was febrile, with a body temperature of 39.5 °C, a heart rate of 110 beats per minute, a respiratory rate of 30 breaths per minute, and a blood pressure of 100/60 mmHg. Physical examination revealed scattered rales on the auscultation of both lungs and mild jaundice. Laboratory investigations showed leukocytosis (white blood cell count:15 × 10^9/L), thrombocytopenia (platelet count: 80 × 10^9/L), elevated liver enzymes (alanine aminotransferase: 200 U/L, aspartate aminotransferase: 250 U/L), and elevated creatinine (150 μmol/L). C-reactive protein was significantly elevated at 120 mg/L. The myositis-specific antibody panel revealed the presence of anti-Ro-52 and low-titer anti-Mi-2β antibodies. Bedside transthoracic echocardiography revealed impaired left ventricular systolic function (ejection fraction, 36%) and mild-to-moderate tricuspid regurgitation. A chest CT scan was performed and showed diffuse bilateral ground-glass opacities and consolidation, consistent with alveolar hemorrhage and ARDS (Figure 1).

### Clinical deterioration

**Figure 2:**
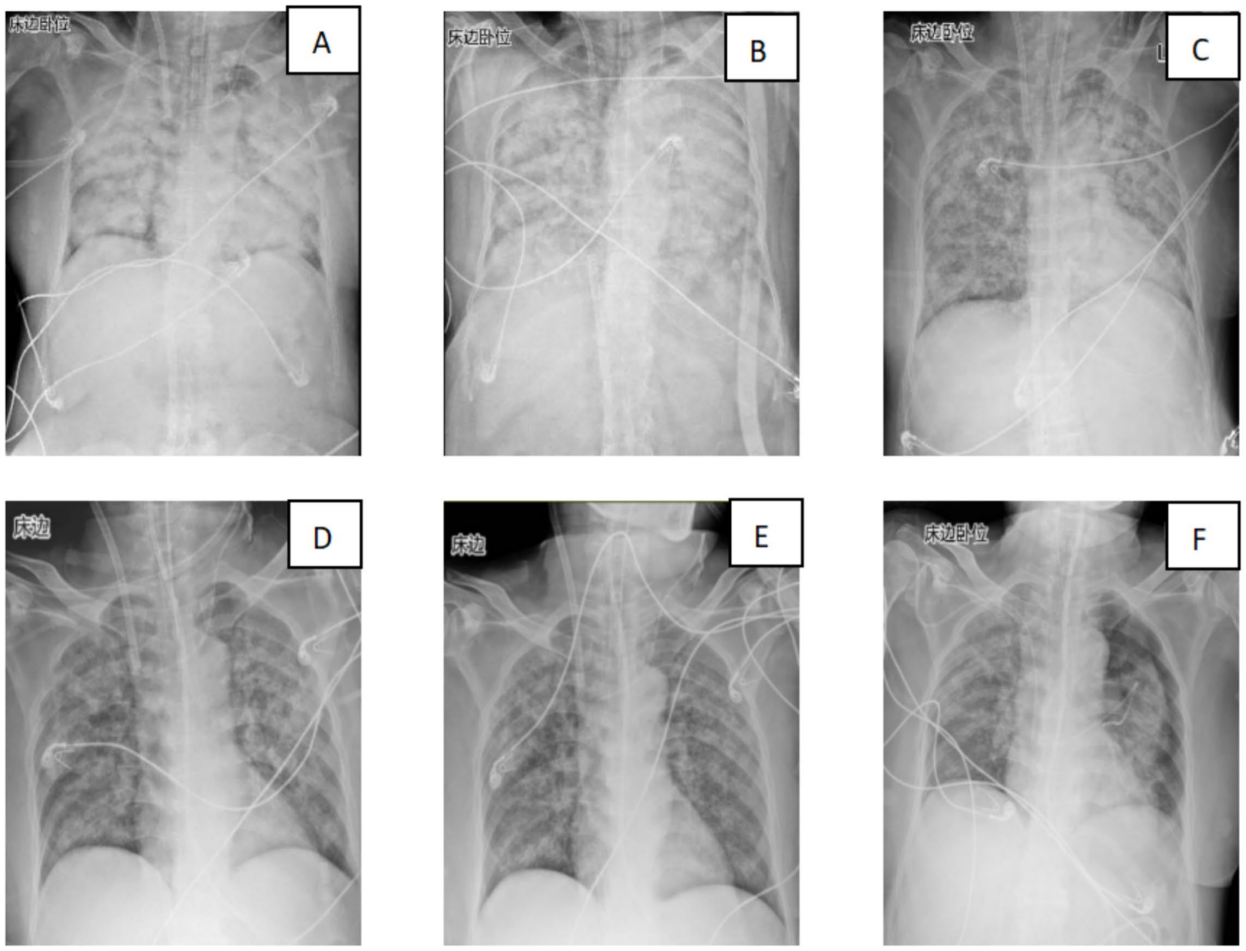
Changes in the patient’s lung imaging (**A**: 29th July; **B**: 30th July 30; C: 31st July; **D**: 2nd August; **E**: 3rd August; and **F**: 11th August).

**Figure 3:**
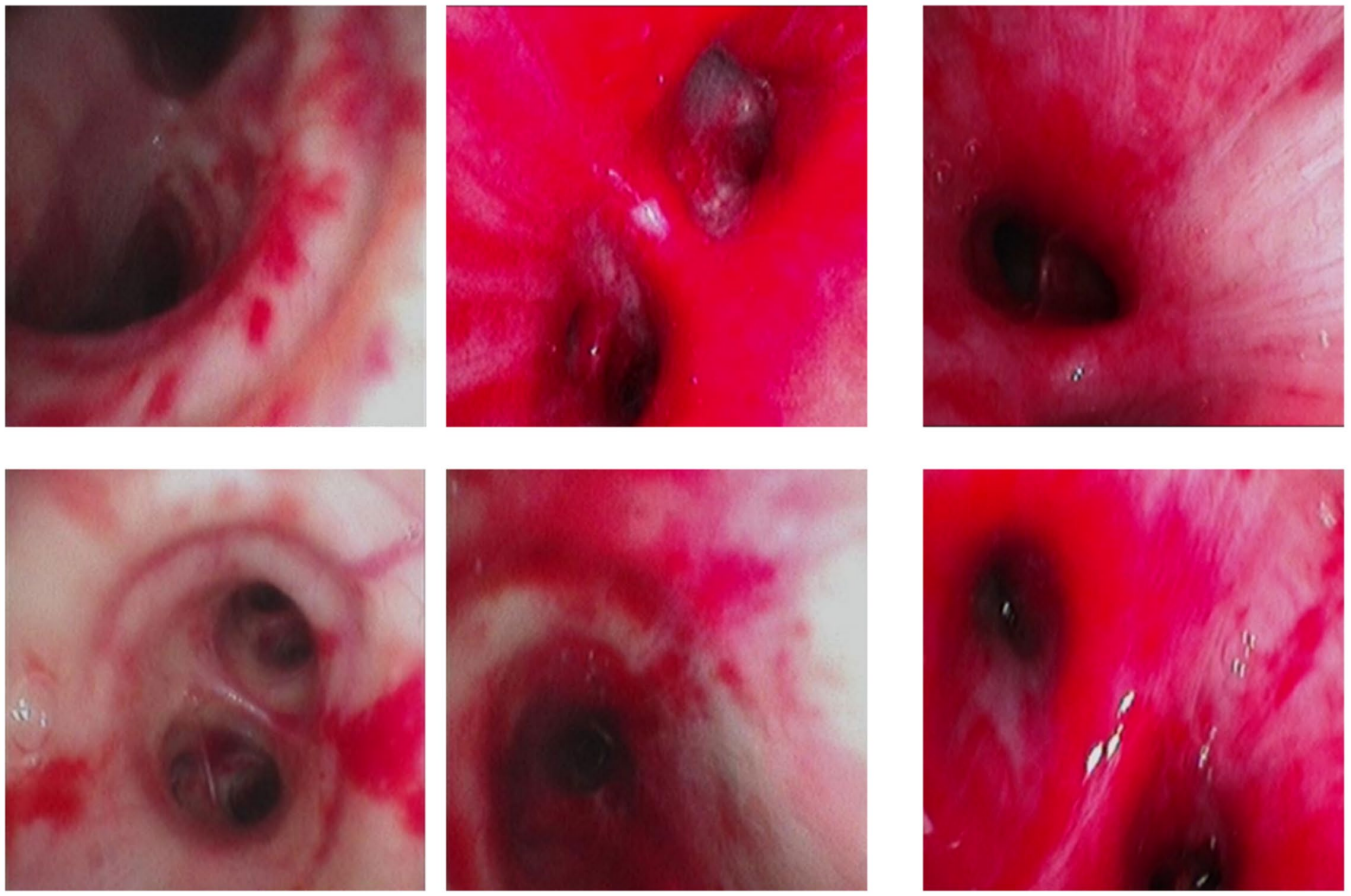
Diffuse redness, swelling, and bleeding from the airway in the patient under bronchoscopy.

**Figure 4:**
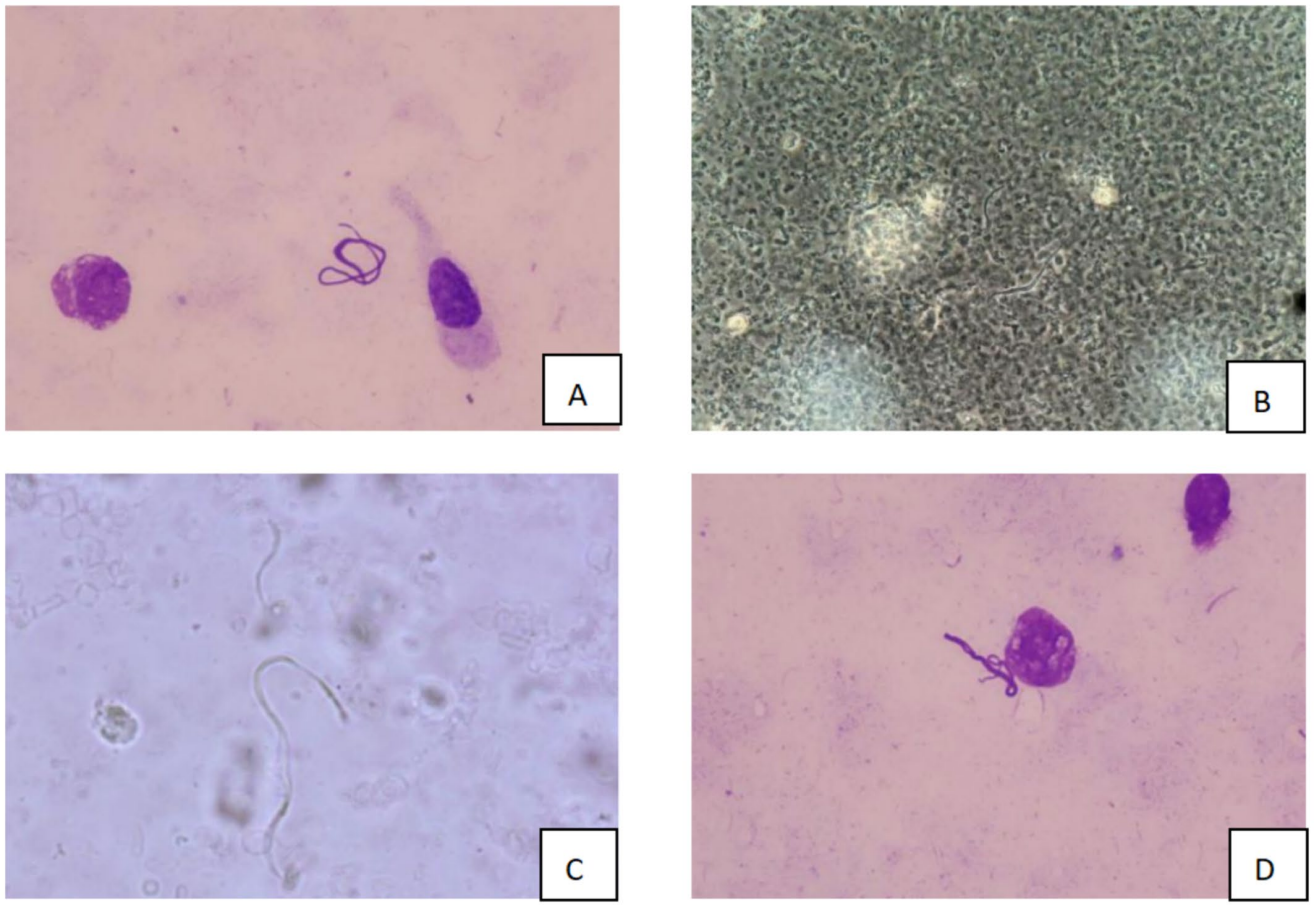
Microscopic examination results of bronchoalveolar lavage fluid (**A/D**: Wright’s staining, 10 × 100 magnification; **B/C**: dark field microscopy, 10 × 40 magnification).

Over the subsequent 24 h, the patient’s condition deteriorated rapidly. Progressive dyspnea developed, and arterial blood gas analysis indicated severe hypoxemia (partial pressure of arterial oxygen/fraction of inspired oxygen ratio [PaO₂/FiO₂] = 107 mmHg) despite optimal mechanical ventilation settings. Chest radiography demonstrated diffuse bilateral infiltrates, consistent with acute respiratory distress syndrome (ARDS) (Figure 2). Bronchoscopy revealed massive endobronchial bleeding, indicative of severe pulmonary hemorrhage (Figure 3). Considering the patient’s rapidly worsening respiratory status and presence of multiorgan dysfunction, a diagnosis of severe leptospirosis with pulmonary hemorrhage was suspected. As this pathogen is not commonly encountered in our hospital, specific antigens for the microscopic agglutination test (MAT) were not available. However, microscopic examination of bronchoalveolar lavage fluid collected via bronchoscopy revealed Leptospira (as shown in Figure 4). After 48 h, polymerase chain reaction (PCR) results from the patient’s blood and alveolar lavage fluid samples confirmed the presence of Leptospira DNA and additional *Mycobacterium chelonae* and *Aspergillus flavus*. The diagnosis of leptospirosis with hemorrhagic lung manifestation was thus confirmed.

## Management

### Initiation of ECMO

Due to refractory hypoxemia and failure to maintain adequate oxygenation with conventional mechanical ventilation, a decision was made to initiate VV-ECMO. Venovenous extracorporeal membrane oxygenation (VV-ECMO) was established via cannulation of the right internal jugular vein and femoral vein. The cannulation procedure involved ultrasound-guided placement of a 21-French (Fr) multistage drainage cannula into the right femoral vein, advanced to a depth of 40 cm at the junction of the inferior vena cava and right atrium. A 17-Fr return cannula was placed in the right internal jugular vein to a depth of 14 cm. The circuit consisted of a Maquet Rotaflow^®^ centrifugal pump with Bioline coating and a Maquet Quadrox^®^ polymethylpentene oxygenator. Initial ECMO settings included a pump speed of 2,500 revolutions per minute (rpm), generating a blood flow rate of 3.5 liters per minute (L/min), and a sweep gas (oxygen) flow rate of 4 L/min. Following ECMO initiation, the patient demonstrated significant improvement in oxygenation, with the ratio of arterial partial pressure of oxygen to fraction of inspired oxygen (PaO₂/FiO₂ ratio) increasing to 250 mmHg.

### Antibiotic therapy

Based on the diagnosis of leptospirosis, intravenous penicillin G (2 million units every 4 h) was immediately administered. Given the polymicrobial infection with *Mycobacterium chelonae* and *Aspergillus flavus*, combination therapy with intravenous amikacin (10–15 mg/kg once daily) and amphotericin B lipid complex (3–5 mg/kg/day) was administered.

### Supportive therapies

In addition to ECMO and antibiotic treatment, the patient received comprehensive supportive care. Continuous renal replacement therapy (CRRT) was initiated due to acute kidney injury. Fluid management was optimized to maintain hemodynamic stability while avoiding fluid overload. Platelet and fresh-frozen plasma transfusions were administered as needed to correct thrombocytopenia and coagulopathy. Given the presentation of pulmonary hemorrhage and the specific autoantibody profile, the differential diagnosis included connective tissue disease-associated vasculitis. This finding warranted antibody-removing therapy; hence, lymphoplasmacyte exchange (LPE) was performed on 31st July, 1st August, and 3rd August. The patient was also placed in the prone position to improve oxygenation and recruit collapsed alveoli. Notably, corticosteroids were not administered due to active pulmonary hemorrhage and concern about exacerbating bleeding, as well as the availability of a more targeted immunomodulatory approach via LPE.

### Clinical course

Over the following days, the patient’s condition gradually improved. The volume of endobronchial bleeding decreased, and the oxygenation index continued to improve. On day 4 of ECMO support, the patient’s lung exudation had decreased compared to before, and oxygenation had begun to improve. On day 8, following a comprehensive assessment of respiratory function, oxygenation status, and overall clinical condition, the decision was made to wean the patient from ECMO. The weaning process was uneventful, and the ECMO circuit was successfully removed. The patient was then gradually weaned off mechanical ventilation over the next 3 days.

### Outcome

The patient was transferred to the general ward on day 15 after admission. Follow-up laboratory tests showed normal liver and kidney function, and the complete blood count had returned to normal. The patient was discharged on day 18 with a prescription for oral doxycycline for a total of 14 days. At the 2-week follow-up visit, the patient reported no residual symptoms, and chest radiography showed complete resolution of pulmonary infiltrates.

## Discussion

Leptospiral pulmonary hemorrhage syndrome (LPHS), the most severe complication of leptospirosis, is characterized by toxin-mediated endothelial damage and cytokine storms that lead to diffuse alveolar hemorrhage (DAH) and refractory hypoxemia, with mortality exceeding 50% (6). Our patient presented with an oxygenation index (PaO₂/FiO₂) of 107 mmHg that persisted despite mechanical ventilation and prone positioning, meeting criteria for severe ARDS (7). Venovenous extracorporeal membrane oxygenation (VV-ECMO) served as a salvage therapy, providing critical time for lung recovery through extracorporeal gas exchange. Notably, our early ECMO initiation within 24 h of mechanical ventilation contrasts with conventional practice. Trejnowska et al. (8) demonstrated that delayed ECMO initiation (>48 h) in LPHS increased mortality 2-fold (OR 2.1, 95% CI 1.3–3.4), aligning with Extracorporeal Life Support Organization (ELSO) guidelines advocating prompt intervention in severe ARDS (9).

The application of ECMO in active pulmonary hemorrhage presents a therapeutic paradox: insufficient anticoagulation increases thrombotic risk (peak D-dimer: 10.40 mg/L), while excessive anticoagulation exacerbates bleeding (10). We implemented a minimal effective anticoagulation strategy (target activated partial thromboplastin time (APTT) 40–50 s) with fibrinogen maintenance >1.5 g/L, combined with topical hemostasis (bronchoscopic lavage + carbazochrome). This approach diverged significantly from published protocols: Suvadeep Sen et al. reported fatalities in both of their two LPHS-ECMO patients, attributing the outcomes to hemorrhage aggravated by conventional anticoagulation (targeting an APTT of 60–80 s) (11). Our patient achieved thromboprophylaxis without new hemorrhagic complications, supporting ELSO’s recommendation for reduced anticoagulation in hemorrhagic infectious disease (12). On the 10th day after admission, follow-up echocardiography showed that the patient’s cardiac function had significantly improved, with a left ventricular ejection fraction (LVEF) of 66% and complete resolution of tricuspid regurgitation. Furthermore, VV-ECMO support improved systemic oxygenation, which in turn mitigated hypoxic pulmonary vasoconstriction and secondary pulmonary hypertension, leading to improved tricuspid regurgitation and reduced right ventricular afterload. This mechanism disrupted the self-perpetuating cycle of hypoxia, pulmonary vasoconstriction, and right heart failure, which was particularly beneficial given the patient’s pre-existing impairment of left ventricular systolic function (13).

Antimicrobial stewardship constituted another cornerstone of recovery. While penicillin remains the gold standard for leptospirosis (>95% susceptibility) (14), Jarisch–Herxheimer reactions may exacerbate pulmonary hemorrhage during treatment initiation (15). Under ECMO support, we administered high-dose penicillin (4.8 million units Q6H vs. conventional 1.6–2.4 million units) without hemodynamic compromise, with alveolar hemorrhage significantly diminishing within 48 h. This corroborates Brett-Major’s meta-analysis, wherein early high-dose penicillin reduced leptospirosis mortality by 79% (RR 0.21, 95% CI 0.08–0.55) (16). The patient subsequently developed a life-threatening polymicrobial co-infection with *Mycobacterium chelonae* and *Aspergillus flavus* in the bronchoalveolar lavage fluid, a consequence of dysbiosis from broad-spectrum antibiotics. To address this and the attendant risk of ECMO circuit biofilm formation (17), we instituted a targeted antimicrobial regimen: amphotericin B lipid complex (for deep tissue penetration) and amikacin (*M. chelonae*). This intervention was critical, as polymicrobial infections have been reported to increase mortality in LPHS to 80% (18), making the insights from this case particularly valuable.

The most innovative intervention was lymphoplasmacyte exchange (LPE). Positive anti-Mi-2β and anti-Ro-52 antibodies suggested autoimmune involvement (e.g., ANCA-associated vasculitis) in lung injury (19). Conventional plasma exchange would deplete coagulation factors and increase bleeding risk. LPE selectively removes pathogenic lymphocytes and antibodies while preserving coagulation function—particularly advantageous for ECMO patients with hemorrhage (19). Post-LPE (three sessions), oxygenation index improved from 107 mmHg to >200 mmHg, and IL-6 normalized from 103.0 pg/mL without bleeding complications. Lymphocytapheresis reduces the IL-6/TNF-*α* load by depleting cytokine-producing lymphocytes, thereby attenuating C5a generation and its downstream endothelial injury pathways (oxidative stress, apoptosis, and barrier dysfunction) (20, 21). To our knowledge, this represents the first successful combination of LPE with ECMO in LPHS with autoimmune seropositivity, offering a novel strategy for critical illnesses at the intersection of infection and immunity.

Critical deviations from conventional management protocols were implemented in this case, with comparative analysis revealing significant associations with improved outcomes. First, VV-ECMO was initiated within 24 h of mechanical ventilation—markedly earlier than the mean 72-h window reported in comparable cohorts (22). This expedited intervention correlated with patient survival, contrasting sharply with the 80% mortality observed under delayed initiation. Second, anticoagulation targeting an APTT of 40–50 s combined with fibrinogen monitoring was used, substantially lower than the standard 60–80 s range. This conservative strategy resulted in zero hemorrhagic complications, compared to a 60% hemorrhage rate associated with conventional intensity anticoagulation. Third, immunomodulation utilizing three sessions of LPE was administered instead of high-dose corticosteroid regimens. This approach demonstrated superior efficacy in normalizing interleukin-6 (IL-6) levels, contrasting with persistent inflammatory responses documented with steroid-based protocols. Compared to Umei N’s ECMO-only approach (reporting 20% survival in LPHS with co-infections) (23), our LPE-ECMO-antimicrobial triad achieved survival where literature predicts <20% success. Weaning followed international consensus: ECMO discontinued when oxygenation index exceeded 200 mmHg with bronchoscopic confirmation of hemorrhage cessation (13).

### Limitations and future directions

Three limitations warrant mention: Diagnostic certainty: Leptospira identification initially relied on morphology rather than the microscopic agglutination test (MAT) or PCR (6); LPE quantification: The efficiency of antibody clearance (e.g., anti-Ro-52) was not measured. Long-term outcomes: Neurological recovery requires extended follow-up. Future studies should establish leptospirosis-ECMO registries to optimize anticoagulation (e.g., thromboelastography-guided) and biomarker-directed LPE indications.

## Conclusion

This case report demonstrates the successful use of VV-ECMO in combination with antibiotics and supportive therapies for a patient with severe leptospirosis complicated by life-threatening pulmonary hemorrhage. Early disease recognition, prompt ECMO initiation, and comprehensive management are key factors in improving prognosis in such patients. Further studies are needed to better define optimal management strategies for leptospirosis-related severe pulmonary hemorrhage and to enhance outcomes in critically ill patients.

## Patient perspective

The patient provided written consent for publication. In follow-up interviews, she expressed gratitude for the “second chance” but highlighted the need for psychological support post-ICU.

## Data Availability

The original contributions presented in the study are included in the article/Supplementary material, further inquiries can be directed to the corresponding authors.
