# Supplementary material for: A case report of leptospirosis complicated by severe pulmonary hemorrhage treated with venovenous extracorporeal membrane oxygenation
Source: Front Med (Lausanne). 2025 Nov 12;12:1696005. doi: 10.3389/fmed.2025.1696005 (PMC12646937; doi:10.3389/fmed.2025.1696005)
Supplement: Supplementary file 1 [file Table_1.docx]

**Table 1 Records of Mechanical Ventilation Parameters (July 29–August 7, 2025)**

| **Monitoring Time** | **Ventilator Model** | **Ventilation Mode** | **FiO₂ (%) (Set\Monitored)** | **RR (bpm) (Set\Spontaneous\Monitored)** | **PEEP (cmH₂O) (Set\Monitored)** | **MV (L/min) (Set\Monitored)** | **VT (mL) (Set\Monitored)** | **Peak (cmH₂O)** | **Plateau Pressure (cmH₂O)** | **Mean Airway Pressure (cmH₂O)** | **Dynamic Compliance (mL/cmH₂O)** |
| --- | --- | --- | --- | --- | --- | --- | --- | --- | --- | --- | --- |
| 2025/7/29 07:00 | Servo - i | VC | 40/40 | 12/12 | 10/10.6 | 3.5/3.7 | 300/305 | 23 | 25.6 | 13.4 | 19.5 |
| 2025/7/30 07:00 | Servo - i | VC | 40/40 | 16/16 | 10/12.4 | 4.7/4.9 | 300/304 | 25.3 | 26.9 | 15.1 | 20.5 |
| 2025/7/31 07:00 | Servo - i | VC | 40/40 | 12/12 | 10/12.5 | 3.5/3.6 | 300/307 | 23.7 | 24.4 | 15.1 | 25 |
| 2025/8/1 07:00 | Servo - i | VC | 40/40 | 12/12 | 10/13.2 | 3.5/3.7 | 300/308 | 21.7 | 21.8 | 15 | 33.5 |
| 2025/8/2 07:00 | Servo - i | VC | 40/40 | 12/12 | 10/13.1 | 3.5/3.6 | 300/303 | 21.6 | 21.4 | 15 | 36.5 |
| 2025/8/3 07:00 | Servo - i | VC | 40/40 | 18/18 | 10/13.1 | 7.3/7.3 | 400/405 | 28.7 | 24.8 | 16.6 | 35.5 |
| 2025/8/4 07:00 | Servo - i | SIMV(VC)+PS | 35/35 | 16/16 | 8/11.6 | 6.3/6.3 | 400/396 | 27.4 | 16.9 | 13.8 | 74 |
| 2025/8/5 07:00 | Servo - i | SIMV(VC)+PS | 35/35 | 15/14 | 7/10.3 | 5.6/6.3 | 400/378 | 16.6 | 8.9 | 10.6 | 48.5 |
| 2025/8/6 07:00 | Servo - i | CPAP | 30/30 | 11 | 5/8.0 | 6.2/6.8 | /599 | 12 | 13.5 | 8.9 | 140 |
| 2025/8/7 07:00 | Servo - i | CPAP | 30/30 | 19 | 5/7.5 | 12.8/13.6 | /733 | 12.8 | 15.4 | 9.5 | 144.5 |

Note: VC = volume - controlled ventilation; SIMV(VC)+PS = synchronized intermittent mandatory ventilation with volume control plus pressure support; CPAP = continuous positive airway pressure; FiO₂ = fraction of inspired oxygen; RR = respiratory rate; PEEP = positive end - expiratory pressure; MV = minute ventilation; VT = tidal volume; Peak = peak airway pressure.

**Table 2 Records of ECMO Parameters and Related Coagulation Function (July 29–August 4, 2025)**

| **Monitoring Time (ECMO Support Duration)** | **ECMO Support Mode** | **Pump Speed (r/min)** | **Blood Flow (L/min)** | **Gas Flow (L/min)** | **FiO₂ (%)** | **SaO₂ (%)** | **SvO₂ (%)** | **Membrane Lung Thrombi (Number)** | **Activated Partial Thromboplastin Time (s)** | **Heparin Loading Dose (ml)** |
| --- | --- | --- | --- | --- | --- | --- | --- | --- | --- | --- |
| 2025/7/29 07:00 (Day 2, Total 18 h) | VV | 2610 | 3.82 | 2.5 | 100 | 99 | 72 | 4 | 39.6 | 0.2 |
| 2025/7/30 07:00 (Day 3, Total 30 h) | VV | 2800 | 4.13 | 2.5 | 100 | 99 | 82 | 7 | 38.4 | 0.2 |
| 2025/7/31 07:00 (Day 4, Total 42 h) | VV | 2710 | 4.08 | 2 | 100 | 99 | 82 | 12 | 35.6 | 0.6 |
| 2025/8/1 07:00 (Day 5, Total 54 h) | VV | 2540 | 3.85 | 2.2 | 90 | 99 | 71 | 13 | 43.7 | 0 |
| 2025/8/2 07:00 (Day 6, Total 66 h) | VV | 2210 | 3.33 | 2 | 60 | 99 | 72 | 13 | 46.3 | 0 |
| 2025/8/3 07:00 (Day 7, Total 78 h) | VV | 2210 | 3.24 | 2 | 0 | 62 | 61 | 14 | 43.4 | 0 |
| 2025/8/4 07:00 (Day 8, Total 90 h) | VV | 1860 | 2.3 | 0 | 0 | 68 | 65 | 15 | 53.3 | 0 |

Note: ECMO = extracorporeal membrane oxygenation; VV = veno - venous ECMO mode; SaO₂ = arterial oxygen saturation; SvO₂ = venous oxygen saturation.
